# The Effects of Exercise Interventions on Ectopic and Subcutaneous Fat in Patients with Type 2 Diabetes Mellitus: A Systematic Review, Meta-Analysis, and Meta-Regression

**DOI:** 10.3390/jcm13175005

**Published:** 2024-08-23

**Authors:** Fatemeh Kazeminasab, Ali Bahrami Kerchi, Nasim Behzadnejad, Saba Belyani, Sara K. Rosenkranz, Reza Bagheri, Fred Dutheil

**Affiliations:** 1 Department of Physical Education and Sports Science, Faculty of Humanities, University of Kashan, Kashan 87317-53153, Iran; 2 Department of Exercise Physiology, Faculty of Sports Science, Isfahan (Khorasgan) Branch, Islamic Azad University, Isfahan P.O. Box 81551-39998, Iran; alibahramikerchi@khuisf.ac.ir; 3 Department of Exercise Physiology, Faculty of Sport Sciences, University of Isfahan, Isfahan P.O. Box 81746-73441, Iran; n.behzadnezhad8884@gmail.com (N.B.); will.fivb@yahoo.com (R.B.); 4 Human Nutrition Program, Department of Human Sciences, The Ohio State University, Columbus, OH 43210, USA; saba.belyani1@gmail.com; 5 Department of Kinesiology and Nutrition Sciences, University of Nevada Las Vegas, Las Vegas, NV 89154, USA; sara.rosenkranz@unlv.edu; 6 University Hospital of Clermont–Ferrand, Université Clermont Auvergne, CNRS, LaPSCo, Physiological and Psychosocial Stress, CHU Clermont–Ferrand, Occupational and Environmental Medicine, F-63000 Clermont–Ferrand, France; fred_dutheil@yahoo.fr

**Keywords:** exercise training, ectopic fat, type 2 diabetes, liver fat, visceral fat, intramuscular fat

## Abstract

**Background/Objectives:** The aim of the present study was to determine the effects of exercise training on ectopic and subcutaneous fat in patients with type 2 diabetes mellitus (T2DM). **Methods:** Web of Science, PubMed, and Scopus were searched for original articles published through November 2023 that included exercise versus control interventions on body mass (BM), liver fat percentage, visceral fat area (VFA), subcutaneous fat area (SFA), and intramuscular fat volume or mass (IMF) in patients with T2DM. Weighted mean differences (WMDs) for liver fat and BM, standardized mean differences (SMDs) for VFA, SFA, and IMF, and 95% confidence intervals (95% CIs) were determined using random-effects models. **Results:** Thirty-six studies comprising 2110 patients with T2DM were included in the present meta-analysis. Exercise training effectively reduced BM [WMD = −2.502 kg, *p* = 0.001], liver fat% [WMD = −1.559%, *p* = 0.030], VFA [SMD = −0.510, *p* = 0.001], and SFA [SMD = −0.413, *p* = 0.001] in comparison to the control. The IMF [SMD = 0.222, *p* = 0.118] remained unchanged compared to the controls. Subgroup analyses showed that the type of exercise, duration, and body mass index (BMI) of participants were sources of heterogeneity. **Conclusions:** The current meta-analysis provides strong evidence that exercise training, particularly aerobic and combined (aerobic and resistance) exercise programs, is effective for reducing BM, VFA, and SFA in patients with T2DM. However, aerobic exercise was more effective for reducing liver fat than combined exercise. The beneficial effects of exercise on VFA and SFA reduction, but not liver fat, are associated with weight loss. These findings highlight the importance of including consistent exercise as a key management component for T2DM and associated ectopic fat deposition, with potential long-term benefits for metabolic health.

## 1. Introduction

The global prevalence of type 2 diabetes mellitus (T2DM) is escalating [1,2], and ectopic fat, inclusive of visceral fat deposition, is known to be a critical contributor to its pathogenesis and progression [3]. It is well established that visceral fat deposition is a risk factor for cardiometabolic diseases [4]. Perhaps less well known, in patients with T2DM, fat accumulates in tissues such as the liver, skeletal muscle, heart, and pancreas, which normally contain only small amounts of fat. This abnormal fat accumulation, known as ectopic fat, can interfere with cellular functions and impair organ function, leading to insulin resistance [5]. Furthermore, ectopic fat deposits may contribute to obesity-related complications and metabolic syndrome by disrupting lipid metabolism [6]. A previous cohort study found that participants who were categorized as having only-ectopic fat obesity had an increased risk of developing T2DM, and this increase in risk was much larger than the risk in participants categorized as only-obesity and only-visceral fat obesity [7]. In addition, people with T2DM have been shown to have more ectopic fat, in particular liver fat, as compared with age- and body mass index-matched participants without T2DM [8]. These studies underscore the importance of ectopic fat in the development and progression of T2DM and highlight the potential benefits of interventions aimed at reducing ectopic fat accumulation.

Exercise training is a well-known strategy for managing T2DM, with beneficial effects on reducing body fat percentage and skeletal muscle lipid content [9,10]. Different types of exercise, including aerobic exercise, resistance training, and combined training (aerobic + resistance exercise), have been shown to be effective in reducing ectopic fat in patients with T2DM [9]. In this regard, a previous systematic review and meta-analysis found that all three types of regular exercise are beneficial for reducing ectopic fat (including visceral fat) in adults with T2DM [11]. It has been proposed that including both aerobic and resistance exercise has advantages for improving hemoglobin A1c (HbAlc) and insulin sensitivity as compared to either type of exercise alone [12]. These previous studies indicate that regular exercise may be a viable approach for decreasing ectopic fat accumulation in patients with T2DM, thereby enhancing insulin sensitivity and overall disease management [12,13].

Some studies have indicated that aerobic exercise may be superior as compared with other types of exercise for decreasing ectopic fat. This is evidenced in a meta-analysis by Sabag and colleagues (2017) that included 24 randomized trials with 1383 patients with T2DM, evaluating the effectiveness of exercise as an intervention for decreasing ectopic fat (visceral adipose tissue [VAT] and liver fat) in patients with T2DM [11]. The results suggested that exercise, especially aerobic exercise, can effectively reduce VAT and liver fat. However, other types of exercise in short-term or longer-term interventions on ectopic and subcutaneous fat, as compared with non-exercise control groups, were not investigated. Additionally, the effects of exercise training on these outcomes in patients with overweight or obesity and T2DM have not been elucidated. Similarly, the relationship between weight loss and the effects of exercise on ectopic and subcutaneous fat in patients with T2DM is unclear. Systematic reviews and meta-analyses have shown that diet and/or exercise are effective for reducing hepatic adiposity in children and adolescents with obesity [14], and for reducing visceral adipose tissue in males and females with overweight [15]. Lastly, a network meta-analysis indicated that high-intensity interval training and aerobic exercise of at least moderate intensity were beneficial for reducing VAT in the general population [16]. However, no systematic review or meta-analysis has examined the effects of exercise interventions on ectopic and subcutaneous fat in patients with T2DM. Therefore, the current systematic review and meta-analysis of randomized controlled trials (RCTs) aimed to elucidate the effects of different exercise modalities, including aerobic, combined (aerobic and resistance), and resistance training, on ectopic (liver, visceral, and intramuscular) and subcutaneous fat in patients with T2DM.

## 2. Methods

### 2.1. Research Question

The present systematic review and meta-analysis was conducted with the aim of determining the effects of exercise interventions on ectopic (including liver fat and visceral fat) and subcutaneous fat in patients with T2DM. This study was performed in accordance with the Preferred Reporting Items for Systematic Reviews and Meta-Analyses (PRISMA) guidelines [17] and the Cochrane Handbook of Systematic Reviews of Interventions. The systematic review and meta-analysis was registered prospectively in the International Prospective Register of Systematic Reviews (PROSPERO) with the identification code CRD42024517106.

### 2.2. Inclusion and Exclusion Criteria

The following inclusion criteria were applied: (1) English language articles; (2) studies of human participants with T2DM; (3) studies where the experimental group underwent an exercise intervention and was compared with a no-intervention control group; (4) RCTs; (5) studies with assessments of liver fat%, visceral fat area (VFA), subcutaneous fat area (SFA), intramuscular fat volume (IMF), pancreatic fat, or myocardial fat with pre- and post-intervention or change scores reported.

Exclusion criteria included (1) studies written in a non-English language; (2) non-original and non-experimental research such as case–control studies, cross-sectional studies, study protocols, conference proceedings, letters to the editor, reviews, and meta-analyses; (3) animal studies; (4) studies where the dietary interventions in the exercise group and the control group were different; and (5) non-randomized studies.

### 2.3. Search Strategy and Retrieval

A comprehensive electronic database search was completed in Scopus, Web of Science, and PubMed. Two reviewers independently identified published articles up to November 2023. The electronic search was limited to articles written in the English language and studies conducted with human participants. There was no limitation on publication dates. The complete search strategy is shown in Supplementary Table S1. Records identified from the searches were imported into EndNote, and all duplicates were removed. Titles, abstracts, and full texts were independently assessed for eligibility by two authors. Any disagreements were resolved by discussion or by involving a third author. Reference lists of all identified studies were manually searched for potentially eligible papers.

### 2.4. Study Selection

Studies were included if the exercise intervention duration was ≥4 weeks. Trials involving supervised and unsupervised progressive aerobic exercise (continuous, interval, or high-intensity interval training [HIIT]) alone, resistance exercise, or combined aerobic and resistance exercise were included. Also, studies with exercise interventions that used a dietary intervention were included only if both groups (intervention “exercise” and comparison “control” groups) were given the same dietary intervention. Studies were included if the liver fat, visceral, or subcutaneous outcomes were quantified by biopsy and histological analysis, magnetic resonance imaging (MRI), proton magnetic resonance spectroscopy (1H-MRS), or computed tomography (CT).

### 2.5. Quality Assessment

The risk of methodological bias was independently assessed by one author and verified by another according to the Physiotherapy Evidence Database (PEDro) scale (Supplementary Table S2). Two items (for non-blinding of participants and therapists) were excluded from the original 11-item scale because participants and intervention providers could not practically be blinded to the assigned exercise conditions during studies. Therefore, study quality was assessed based on 9 items (eligibility criteria, random allocation of participants, assessed outcomes in 85% of participants, baseline comparison, allocation concealment, intention-to-treat analysis, reporting of statistical comparisons between groups, and point estimates and variability statistics).

### 2.6. Data Extraction

The following information was collected: author name(s), publication year, participant characteristics (sample size, biological sex, health condition, age, and body mass index [BMI]), study design (exercise type, duration of exercise, and dietary intake), measurement methodologies, and pre- and post-test data for the included outcome variables. Means and standard deviations (SDs) for all primary and secondary outcomes were collated into a single spreadsheet and sorted by outcome for further analysis. In cases where the means and standard deviations (SDs) were not clearly provided, the SDs were calculated using alternative measures such as standard errors of means (SEMs), means and interquartile ranges (IQRs), or medians and IQRs [18,19].

### 2.7. Statistical Analysis

Meta-analyses were performed using Comprehensive Meta-analysis (CMA) software (version 2.0, Biostat Inc., Englewood, NJ, USA) to calculate standardized mean differences (SMDs) or weighted mean differences (WMDs) and 95% confidence intervals (CIs) for primary and secondary outcomes using random-effects models. The units of measurement of liver fat (%) and body mass (BM; kg) were the same, so for these outcomes, WMD was used. The units of measurement of visceral fat area (VFA) (cm^2^, cm^3^, or gr), subcutaneous fat area (SFA) (cm^2^, or cm^3^), and intramuscular fat (IMF) (cm^2^, or gr) were different across the included studies; therefore, SMD was used for these outcomes. Effect sizes were calculated to compare the effects of exercise groups versus control groups (unexercised participants) on visceral, subcutaneous, or intramuscular fat. Effect sizes were evaluated as follows: 0–0.2 as very small, 0.2–0.5 as small, 0.5–0.8 as moderate, or >0.8 as large [20].

Heterogeneity was assessed using the I^2^ statistic, and evaluation of heterogeneity was conducted according to Cochrane guidelines as follows: 25% as low, 50% as moderate, and 75% as high heterogeneity [21]. The significance level was *p* < 0.05.

Subgroup analyses were performed according to the type of exercise (aerobic, resistance, or combined), intervention duration (short-term interventions ≤ 12 weeks, or long-term interventions > 12 weeks), and participant BMIs (25–30 kg·m^−2^ as having overweight, or >30 kg·m^−2^ as having obesity). The only diagnosed condition included in the studies was T2DM; therefore, there was no subgroup analysis by health condition. Additionally, univariate meta-regression analyses of BM and ectopic fat (liver fat, VFA, and intramuscular fat), or SFA, were conducted comparing exercise versus control.

### 2.8. Sensitivity Analysis

Sensitivity analyses were also conducted for all outcomes using the “remove 1” technique. This procedure assessed whether individual studies had a disproportionate impact on the results of the meta-analyses.

### 2.9. Publication Bias

Publication bias was detected through the visual interpretation of funnel plots. If publication bias was present, Egger’s tests were used as a confirmatory test. Significant publication bias was deemed apparent if *p* < 0.1 [22].

## 3. Results

### 3.1. Included Studies

**Figure 1:**
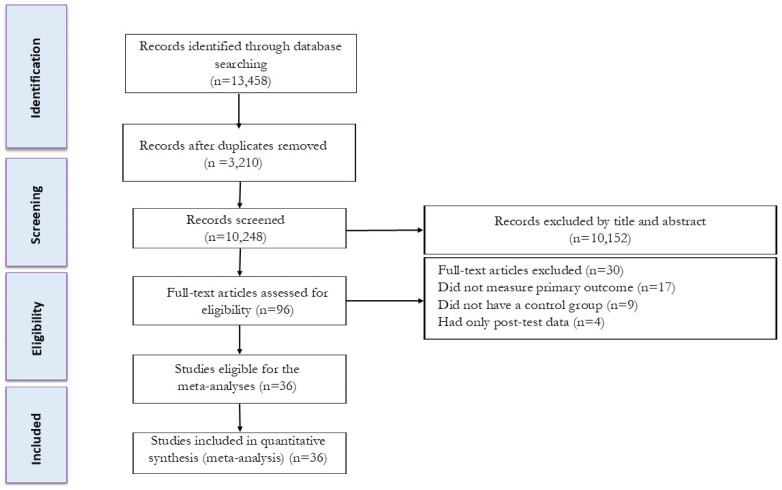
Flow diagram of systematic literature search.

Our initial search strategy identified 5720 records from Scopus, 4201 records from Web of Science, and 3537 records from PubMed. After eliminating duplicate records (3210) and screening the titles and abstracts (initial screening), 66 studies were retrieved for a more detailed appraisal of the full texts (secondary screening). Thirty studies were excluded after reviewing the full text for the following reasons: (A) seventeen did not measure primary outcomes (liver fat, VFA, intramuscular fat, or SFA); (B) nine did not have a control group; and (C) four had only post-test data. When there were missing data, corresponding authors were contacted; however, none provided the necessary information for these studies to be included. A total of 36 studies, inclusive of 45 intervention groups, were included in the present systematic review and meta-analysis. A detailed flow diagram of the systematic literature search is presented in Figure 1.

### 3.2. Participant Characteristics

A total of 2110 patients with T2DM were included, with sample sizes ranging from 16 [23] to 251 [24]. The mean ages of participants ranged from 45 [23,25] to 72 years [26], and the mean BMIs of participants ranged from 24 [27] to 37 kg·m^−2^ [28,29]. The mean age of exercised participants was 57.4 ± 5.5 years, and the mean age of control groups was 57.8 ± 5.3 years. The mean BMI of exercised participants was 30.63 ± 3.74 kg·m^−2^, and the mean BMI of control groups was 30.46 ± 3.55 kg·m^−2^. Both males and females were included in twenty-five studies [1,24,25,26,27,28,29,30,31,32,33,34,35,36,37,38,39,40,41,42,43,44,45,46,47], females only in ten studies [48,49,50,51,52,53,54,55,56,57], and males only in one study [23]. All patients with T2DM were either overweight or obese according to their BMI. Table 1 presents the full details of participant characteristics.

### 3.3. Intervention Characteristics

Intervention durations ranged from 8 [1,23,30,35] to 52 weeks [26,42], with 12-week durations in the majority of studies [25,28,33,36,43,44,48,51,52,53,54,55,56,57]. In four studies, resistance exercise was compared with a control [33,34,42,55], fifteen studies compared aerobic exercise vs. control [23,27,29,30,35,36,38,40,42,47,48,52,53,56,57], and seven studies compared combined exercise vs. control [26,31,32,37,43,44,45]. Twelve studies used more than one type of exercise protocol as separate interventions [1,24,28,35,39,41,46,49,50,51,53,54]. Exercise sessions were performed 2 [35] to 7 times per week [34,47,53], with 3 sessions being the most common (*n* = 22) [1,24,25,27,28,29,30,31,32,33,36,37,38,42,43,44,46,49,52,55,56,57].

The duration of each session of resistance exercise ranged from 30 [34] to 120 min [55], and one study did not mention session duration [33]. The intensity of each session of resistance exercise ranged from 40 to 50% of one-repetition maximum (1RM) [54,55] to 80% of 1RM [42].

The duration of each session of aerobic exercise ranged from 15 min [24] to 120 min [53]. The intensity of each session of aerobic exercise ranged from 50% of Wpeak (peak power output) [46] to 95% of Wpeak [46]. The duration of each session of combined exercise varied from 50 min [41] to 90 min [26], and four studies did not mention session duration [24,31,32,37]. The intensity of each session of resistance exercise in combined interventions ranged from 50% 1RM [31,32,37] to 85% 1RM [26], and the intensity of each session of aerobic exercise in combined interventions ranged from 60% of maximum heart rate (MHR) [24,45,49] to 100% of MHR [41]. The detailed intervention characteristics are presented in Table 2.

### 3.4. Meta-Analysis

#### 3.4.1. Exercise vs. Control

##### Body Mass

**Figure 2:**
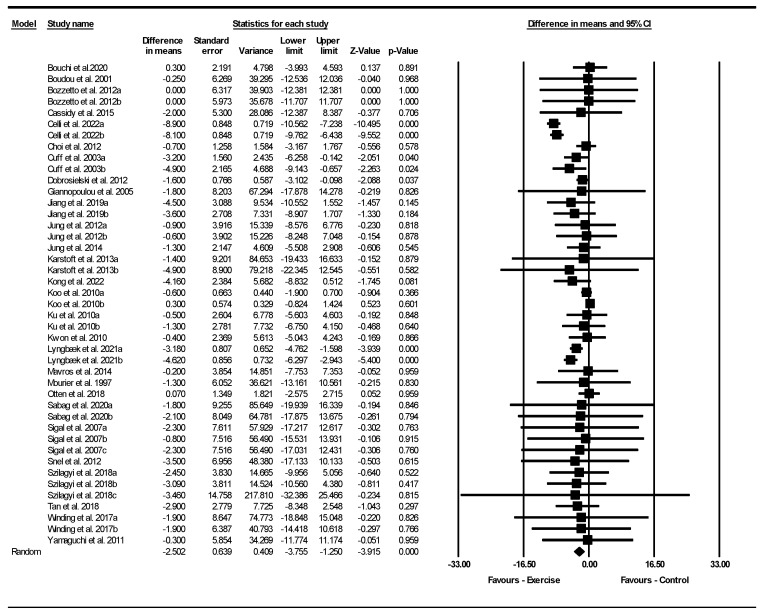
Forest plot of the effects of exercise training vs. control on body weight. Data are reported as WMD (kg) (95% confidence limits). WMD, weighted mean difference [23,24,25,26,28,29,34,35,36,37,38,39,40,41,42,43,45,46,47,48,49,50,51,52,53,54,55,57].

Based on 43 intervention arms with 1815 participants, exercise effectively reduced BM [WMD = −2.50 kg (95% CI: −3.75 to −1.25), *p* = 0.001] when compared to control groups (Figure 2).

Subgroup analyses revealed significant reductions in BM for aerobic [WMD = −0.68 kg (95% CI: −1.39 to 0.01), *p* = 0.049, 26 interventions] and combined interventions [WMD = −4.16 kg (95% CI: −6.35 to −1.97), *p* = 0.001, 12 interventions], but not for resistance exercise [WMD = −0.17 kg (95% CI: −2.66 to 2.31), *p* = 0.880, 5 interventions], when compared with a control group.

In addition, subgroup analyses revealed significant reductions in BM for long-term > 12 weeks [WMD = −4.13 kg (95% CI: −5.84 to −2.41), *p* = 0.001, 19 interventions] but not for short-term interventions ≤ 12 weeks [WMD = −0.33 kg (95% CI: −1.03 to 0.37), *p* = 0.350, 24 interventions] when compared with a control group.

Subgroup analyses by BMI category indicated a significant reduction in BM for patients with obesity [WMD = −3.65 kg (95% CI: −5.40 to −1.91), *p* = 0.001, 23 interventions], but not for patients with overweight [WMD = −0.49 kg (95% CI: −1.20 to 0.21), *p* = 0.170, 20 interventions] when compared with a control group.

There was significant heterogeneity among the included studies (I^2^ = 72.66%, *p* = 0.001). Visual interpretation of funnel plots and Egger’s test (*p* = 0.810) results did not show publication bias. Sensitivity analysis performed by removing individual studies showed that the significance and direction of the results were not disproportionately affected by any individual study.

##### Liver Fat (%)

**Figure 3:**
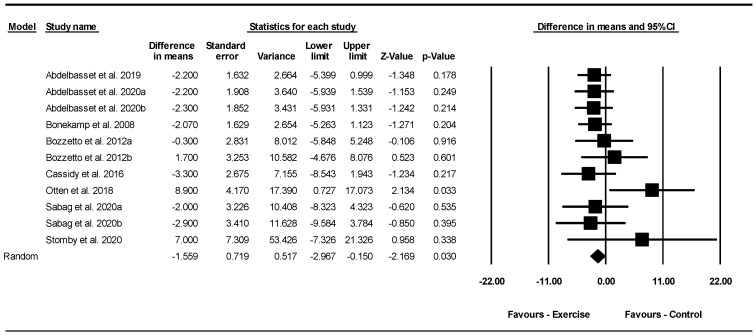
Forest plot of the effects of exercise training vs. control on liver fat (%). Data are reported as WMD (95% confidence limits). WMD, weighted mean difference [1,28,30,32,35,36,43,44].

Based on 11 intervention arms with 279 participants, exercise effectively reduced liver fat [WMD = −1.55% (95% CI: −2.96 to −0.15), *p* = 0.030] when compared to control groups (Figure 3).

Subgroup analyses revealed significant reductions in liver fat for aerobic [WMD = −1.94% (95% CI: −3.55 to −0.33), *p* = 0.010, 8 interventions] but not for combined exercise [WMD = 3.48% (95% CI: −5.01 to 11.98), *p* = 0.420, 3 interventions] when compared with a control group. For liver fat outcomes, eight studies examined the effect of aerobic exercise, three studies examined the effect of combined exercise on liver fat, and no studies investigated the effect of resistance training. Due to the small number of studies available for this outcome, subgroup analyses for training duration and BMI were not possible.

There was no significant heterogeneity among the included studies (I^2^ = 0.00%, *p* = 0.440). Visual interpretation of funnel plots and Egger’s test (*p* = 0.020) results showed publication bias. Sensitivity analysis, conducted by excluding each study individually, revealed that after removing the Abdelbasset et al. 2019, Abdelbasset et al. 2020a, Abdelbasset et al. 2020b, Casidy et al. 2016, Sabag et al. 2020a, and Sabag et al. 2020b studies [1,28,30,36], there were changes in the effect sizes and significance of the results (WMD = −1.33%, *p* = 0.110), (WMD = −1.37%, *p* = 0.090), (WMD = −1.35%, *p* = 0.110), (WMD = −1.37%, *p* = 0.070), (WMD = −1.46%, *p* = 0.060), (WMD = −1.42%, *p* = 0.060), respectively, while the direction of the results remained consistent.

Meta-regression was performed to determine whether BM loss influenced the effects of exercise on liver fat, indicating no significant correlation (coefficient: −1.84; 95% CI: −4.69 to 1.00, *p* = 0.200). This result suggested that there was no significant moderating effect of BM loss.

##### Visceral Fat Area (VFA)

**Figure 4:**
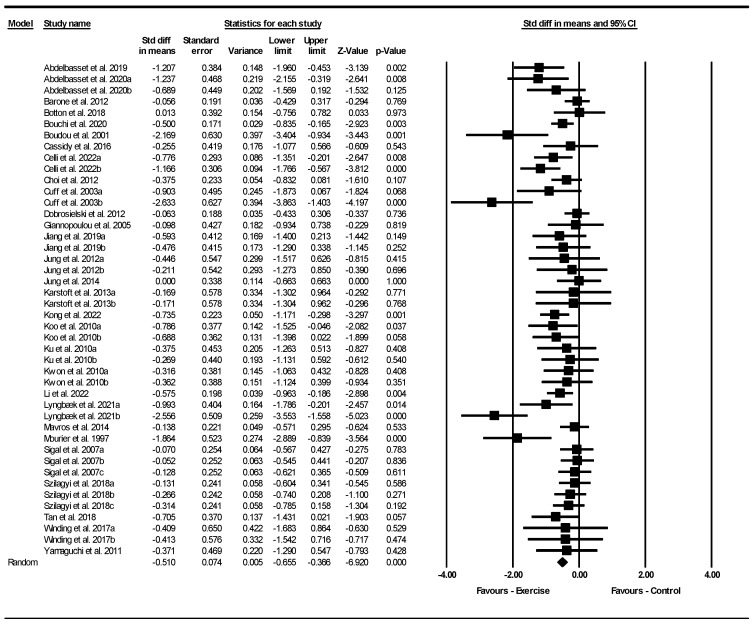
Forest plot of the effects of exercise training vs. control on visceral fat area (VFA). Data are reported as SMD (95% confidence limits). SMD, standardized mean difference [1,23,24,25,26,27,30,31,33,34,36,37,38,39,40,41,42,45,46,47,48,49,50,51,52,53,54,55,56,57].

Based on 44 intervention arms with 1950 participants, exercise effectively reduced VFA [SMD = −0.51 (95% CI: −0.65 to −0.36), *p* = 0.001] with a moderate effect size when compared to control groups (Figure 4).

Subgroup analyses revealed significant reductions in VFA for aerobic exercise [SMD = −0.55 (95% CI: −0.71 to −0.39), *p* = 0.001, 27 interventions] with a moderate effect size, combined training [SMD = −0.65 (95% CI: −1.02 to −0.28), *p* = 0.001, 11 interventions] with a moderate effect size, and resistance exercise [SMD = −0.27 (95% CI: −0.48 to −0.07), *p* = 0.009, 6 interventions] with a small effect size, when compared with a control group.

In addition, subgroup analyses revealed significant reductions in VFA for long-term interventions > 12 weeks [SMD = −0.49 (95% CI: −0.69 to −0.29), *p* = 0.001, 22 interventions], with a small to moderate effect size, as well as short-term interventions ≤ 12 weeks [SMD = −0.54 (95% CI: −0.74 to −0.34), *p* = 0.001, 22 interventions] with a moderate effect size, when compared with a control group.

Subgroup analyses by BMI indicated significant reductions in VFA for patients with obesity [SMD = −0.60 (95% CI: −0.85 to −0.34), *p* = 0.001, 21 interventions] with a moderate effect size, and patients with overweight [SMD = −0.49 (95% CI: −0.63 to −0.35), *p* = 0.001, 23 interventions] with a small to moderate effect size when compared with a control group.

There was significant heterogeneity among the included studies (I^2^ = 51.64%, *p* = 0.001). Visual interpretation of funnel plots and Egger’s test (*p* = 0.002) results also showed publication bias. Sensitivity analysis performed by removing individual studies showed that the effect sizes, significance, and direction of the results did not change.

Meta-regression was used to determine whether BM loss influenced the effects of exercise on VFA, indicating a significant correlation (coefficient: −0.15; 95% CI: −0.30 to 0.00, *p* = 0.040). This result suggested that there was a significant moderating effect of BM loss.

##### Subcutaneous Fat Area (SFA)

**Figure 5:**
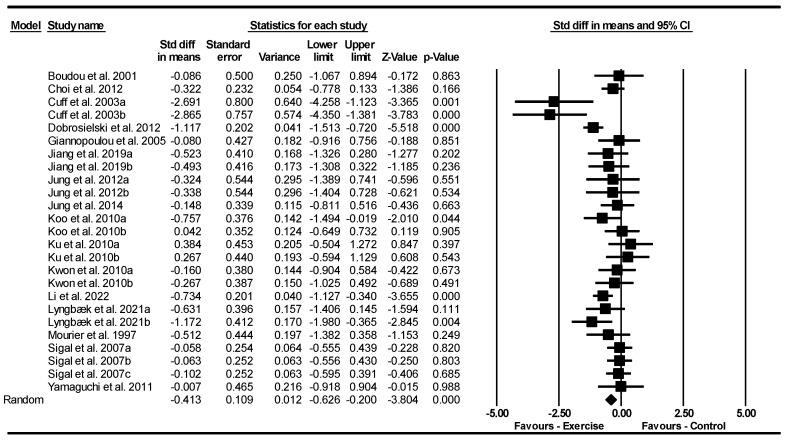
Forest plot of the effects of exercise training vs. control on subcutaneous fat area (SFA). Data are reported as SMD (95% confidence limits). SMD, standardized mean difference [23,24,25,27,37,38,41,47,48,49,50,51,52,53,54,55,56].

Based on 25 intervention arms with 1950 participants, exercise effectively reduced SFA [SMD = −0.41 (95% CI: −0.62 to −0.20), *p* = 0.001] with a small effect size when compared to control groups (Figure 5).

Subgroup analyses revealed significant reductions in SFA for aerobic exercise [SMD = −0.35 (95% CI: −0.55 to −0.14), *p* = 0.001, 16 interventions] with a small effect size, and combined training [SMD = −0.85 (95% CI: −1.43 to −0.26), *p* = 0.004, 6 interventions] with a large effect size, but not for resistance exercise [SMD = −0.009 (95% CI: −0.38 to 0.36), *p* = 0.960, 3 interventions] when compared with a control group.

In addition, subgroup analyses revealed significant reductions in SFA for long-term interventions > 12 weeks [SMD = −0.72 (95% CI: −1.09 to −0.34), *p* = 0.001, 11 interventions] with a moderate effect size, but not for short-term interventions ≤ 12 weeks [SMD = −0.19 (95% CI: −0.39 to 0.01), *p* = 0.067, 14 interventions] with a very small to small effect when compared with a control group.

Subgroup analyses by BMI indicated significant reductions in SFA for patients with obesity [SMD = −0.71 (95% CI: −1.15 to −0.26), *p* = 0.002, 10 interventions] with a moderate effect size, and patients with overweight [SMD = −0.32 (95% CI: −0.50 to −0.14), *p* = 0.001, 15 interventions] with a small effect size when compared with a control group.

There was significant heterogeneity among the included studies (I^2^ = 55.26%, *p* = 0.001). The visual interpretation of funnel plots and Egger’s test (*p* = 0.830) results did not show publication bias. Sensitivity analysis performed by removing individual studies showed that the effect sizes, significance, and direction of the results did not change.

Meta-regression was used to determine whether BM loss influenced the effects of exercise on SFA, indicating a significant correlation (coefficient: −0.26; 95% CI: −0.49 to −0.03; *p* = 0.020). This result suggested that there was a significant moderating effect of BM loss.

##### Intramuscular Fat

**Figure 6:**
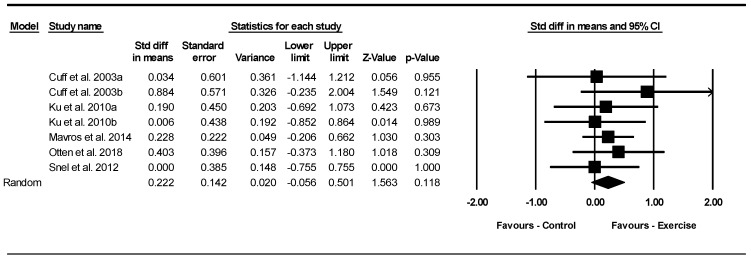
Forest plot of the effects of exercise training vs. control on intramuscular fat. Data are reported as SMD (95% confidence limits). SMD, standardized mean difference [29,42,43,49,54].

Based on seven intervention arms with 208 participants, exercise did not change intramuscular fat [SMD = 0.22 (95% CI: −0.05 to 0.50), *p* = 0.110] with a small effect size when compared to control groups (Figure 6).

Subgroup analyses revealed no significant differences in intramuscular fat for aerobic exercise [SMD = 0.009 (95% CI: −0.50 to 0.51), *p* = 0.970, 3 interventions] with a very small effect size, combined training [SMD = −0.56 (95% CI: −0.07 to 1.19), *p* = 0.086, 2 interventions] with a moderate effect size, or resistance exercise [SMD = 0.22 (95% CI: −0.16 to 0.61), *p* = 0.260, 2 interventions] with a small effect size when compared with a control group.

In addition, subgroup analyses revealed no significant differences in intramuscular fat for long-term interventions > 12 weeks [SMD = 0.22 (95% CI: −0.11 to 0.56), *p* = 0.190, 4 interventions] with a small effect size or short-term interventions ≤ 12 weeks [SMD = 0.21 (95% CI: −0.26 to 0.69), *p* = 0.380, 3 interventions] with a small effect size, when compared with a control group.

Subgroup analyses by BMI indicated no significant differences in intramuscular fat for patients with obesity [SMD = 0.25 (95% CI: −0.05 to 0.56), *p* = 0.110, 5 interventions] with a small effect size, or patients with overweight [SMD = 0.09 (95% CI: −0.52 to 0.71), *p* = 0.760, 2 interventions] with a very small effect size when compared with a control group.

There was no significant heterogeneity among the included studies (I^2^ = 0.00%, *p* = 0.890). The visual interpretation of funnel plots and Egger’s test (*p* = 0.750) results also did not show publication bias. Sensitivity analysis performed by removing individual studies showed that the effect sizes, significance, and direction of the results did not change.

Meta-regression determined whether BM loss influenced the effects of exercise on intramuscular fat, indicating no significant correlation (coefficient: 0.26; 95% CI: −0.06 to 0.58; *p* = 0.110). This result suggested that there was no significant moderating effect of BM loss.

### 3.5. Quality Assessment

The methodological quality of individual studies was evaluated using the PEDro scale, with scores ranging from 6 to 8 out of a maximum of 9 points. Four studies had a score of 8, ten studies had scores of 7, seventeen scored 6, and five studies scored 5. Most of the studies received lower scores due to three evaluation criteria (concealed allocation, blinding of all assessors, and intention-to-treat analysis). The details of the quality assessment are provided in Supplementary Table S1.

## 4. Discussion

The results of the current systematic review, meta-analysis, and meta-regression shed light on the effects of exercise interventions for reducing ectopic (including visceral fat) and subcutaneous fat in patients with T2DM. The results indicated that exercise interventions were generally effective for reducing various measures of ectopic fat, including liver fat and VFA, but not intramuscular fat. Additionally, exercise was effective for reducing SFA when compared to control groups. Aerobic and combined exercise emerged as potent interventions for managing ectopic fat, particularly aerobic exercise, highlighting the potential effectiveness of these types of interventions for enhancing metabolic health among individuals with T2DM. Furthermore, the duration of exercise interventions appears to be important, with long-term interventions (>12 weeks) demonstrating more pronounced reductions in BM and SFA compared to shorter interventions (≤12 weeks). However, the difference between the effects of long and short-term interventions for VFA reduction was not large, and both durations showed significantly larger decreases as compared with a control, underscoring the importance of sustained engagement in exercise for BM management. These comprehensive findings provide practical, translatable, and contemporary knowledge for physicians and sports medicine experts to design exercise training interventions to improve metabolic health in T2DM patients.

There is a large body of evidence on different types of exercise and their effects on fat accumulation in individuals with T2DM [58]. One previous study determined the effects of aerobic, resistance, and combined training on body composition. The results showed that only combined exercise led to a significant decrease in BMI post-intervention. Also, both resistance and combined exercise resulted in significant reductions in body fat and VFA, with combined exercise showing a larger effect [59]. In comparison, our meta-analysis showed that combined exercise had a slightly larger effect on VFA compared to aerobic exercise, with aerobic exercise being more effective than resistance training. This aligns with a previous meta-analysis in healthy adults and youth with obesity, which concluded that programs combining resistance and aerobic training were the most effective for reducing VFA [60]. Another meta-analysis highlighted the effectiveness of exercise, particularly aerobic exercise, in reducing VFA and liver fat in adults with T2DM [11].

Aerobic exercise primarily focuses on improving cardiorespiratory fitness and is effective in reducing total and regional SAT and VAT [61,62], while resistance exercise targets muscle strength and mass, leading to an increase in skeletal muscle mass 63]. Research has shown that regular resistance exercise can stimulate lipolytic activity, contributing to efficient reductions in adipose tissue mass, especially in women with obesity [63]. Additionally, resistance exercise has been found to positively affect the lipolysis pathway by promoting lipid degradation and reducing fat mass [64]. However, the current results indicate limited effects on BM and ectopic fat following resistance exercise alone. The only significant effect from the subgroup analysis based on exercise type was for VFA, and the effect size for resistance exercise was small. In alignment with the current results, previous research has shown that progressive resistance exercise combined with BM loss did not lead to greater improvements in the fatty liver index when compared to BM loss alone in older adults with T2DM [65].

Previous research has indicated that both aerobic and resistance exercise have shown effectiveness in improving body composition and reducing body fat percentage, albeit through different physiological mechanisms [66]. Accordingly, a combined exercise intervention consisting of both aerobic and resistance exercise may provide the most comprehensive approach to body fat reduction by targeting both cardiovascular fitness and muscle strength [60,67]. Studies suggest that a combination of aerobic and resistance exercise leads to greater improvements in insulin sensitivity, glucose metabolism, and lipid profiles compared to either exercise modality alone [68,69]. However, some of our results did not show a benefit for combined interventions. Subgroup analyses revealed significant reductions in liver fat % only for aerobic exercise. Aerobic exercise, in particular, has been shown to have a significant impact on liver fat, demonstrating reduced liver fat content and improved liver function following aerobic exercise interventions [70]. Similar to the current results, an interventional study indicated that in adolescent girls with obesity, aerobic but not resistance exercise is effective for reducing liver fat and visceral adiposity while improving insulin sensitivity, independent of BM loss or calorie restriction [28]. Additionally, aerobic exercise was more effective than resistance exercise in reducing VFA in overweight or obese adults with T2DM [28]. However, the optimal type, intensity, and duration of exercise in managing liver fat in patients with T2DM remain uncertain [71]. 

It is important to highlight liver fat results, as liver fat deposition has been linked to metabolic derangements and the development of insulin resistance in T2DM patients [72]. Our results demonstrate that aerobic exercise is effective for reducing liver fat, while resistance exercise interventions have limited effects on ectopic depots. This suggests that aerobic exercise may be a more suitable intervention for individuals with T2DM who have high levels of liver fat. However, a combination of aerobic and resistance exercise may still be beneficial for overall improvements in ectopic fat, body composition, insulin sensitivity, and lipid profiles.

The current findings on the effects of exercise on SFA also indicate superiority for combined exercise with a large effect size. In contrast, previous research has suggested the superiority of aerobic exercise for reducing SFA, especially total and regional SAT [73]. This discrepancy could be attributed to various factors, including the specific types of exercise included in the ‘combined exercise’ category, the durations and intensities of the interventions, and the characteristics of the study participants.

It is worth noting that there was only one study regarding the effect of exercise on pancreatic fat, which we therefore could not include in the analysis. Research has shown that six months of moderate-intensity aerobic exercise effectively reduces pancreatic fat content, which is crucial for preserving beta-cell function and improving hemoglobin A1C (HbA1c) levels [27]. Additionally, short-term exercise, whether sprint interval training (SIT) or moderate-intensity continuous training (MICT), decreased pancreatic fat in both healthy individuals and those with prediabetes or T2DM, leading to improved beta-cell function [74]. Furthermore, long-term exercise has been found to reduce islet fibrosis, preserve pancreatic islet structure, and maintain beta-cell mass through anti-inflammatory and anti-fibrotic actions, indicating additional benefits for preventing and treating T2DM [75].

Exercise has been shown to have varying effects on myocardial triglyceride levels in different populations. In healthy individuals who are overweight, 12 weeks of combined aerobic and resistance exercise led to reduced cardiac lipid content and improved cardiac function [76]. Conversely, endurance athletes exhibited lower myocardial triglyceride content compared to healthy controls, indicating a potential association between exercise and decreased triglyceride levels in heart tissue [77]. In patients with T2DM, exercise training resulted in improved cardiovascular markers and insulin sensitivity but surprisingly did not lead to a decrease in cardiac lipid content [78]. These results suggest that while exercise can positively impact cardiac function in T2DM patients, a reduction in myocardial triglyceride levels may not be a prerequisite for these improvements, which is aligned with the one study of myocardial ectopic fat we evaluated [76].

Overall, results did not indicate significant changes in intramuscular fat, with some studies indicating an increase following exercise training [43,49]. An interventional study indicated that intramuscular triacylglycerol content increased twofold in response to the 6 months of exercise training [79]. Controversially, one systematic review and meta-analysis did not show a significant effect of exercise on intramuscular fat [80]. However, this can be explained by considering the role of intramuscular triacylglycerol as a dynamic fat-storage depot and a source of energy during exercise [81], contributing up to 20% of total energy turnover during exercise, depending on factors like diet, biological sex, and exercise type [82]. This means that during periods of physical activity, the body might increase intramuscular triacylglycerol stores in anticipation of energy requirements, indicating a positive training adaptation [83,84]. This would be particularly relevant in T2DM patients who have energy dysregulation due to insulin resistance [85,86].

In contrast to liver fat and intramuscular fat, the meta-regression analyses included in the current study showed a significant correlation between BM loss and the effects of exercise on VFA and SFA. This suggests that greater BM loss is associated with more significant reductions in VFA and SFA. These correlations can be attributed to several mechanisms. Firstly, exercise interventions improve adipose tissue function and metabolism, reducing adipocyte size while enhancing insulin sensitivity, which initially leads to decreased VFA and SFA [87,88]. Additionally, exercise promotes lipid mobilization and utilization, with subcutaneous depots being utilized for energy during physical activity [89].

Despite the fact that our meta-regression did not show any significant correlation between BM and the effects of exercise on most outcomes, exercise-induced BM loss is associated with favorable hormonal adaptations, including reduced insulin, leptin, and cortisol levels, while increasing adiponectin, which regulates fat distribution and metabolism [90,91]. Exercise also exerts anti-inflammatory effects [92], reducing visceral fat-associated inflammation and improving metabolic health [93]. Overall, the beneficial effects of exercise on visceral fat depots suggest that body fat management is a crucial aspect of improving morbidity in T2DM and its complications.

### Strengths and Limitations

The present study is a novel addition to the existing body of literature on exercise training in patients with T2DM. Some novel aspects include the inclusion of SFA as an outcome, as well as subgroup analyses based on the types of exercise, durations, and participant BMIs. However, this study has several limitations that may affect the interpretation of the results. First, there was significant heterogeneity for most of the outcomes and publication bias for some outcomes. This heterogeneity may be due to study design, sample sizes, exercise protocols, and intervention durations, making the true differences between exercise interventions and controls difficult to interpret. Second, in some cases, results were affected by individual studies. Third, for some outcomes, there were few studies available, preventing subgroup analyses. Finally, for specific types of ectopic fat, particularly pancreatic and myocardial fat depots, there was only one study available, precluding inclusion in the meta-analysis.

## 5. Conclusions

In conclusion, the current meta-analysis provides evidence that exercise interventions, particularly combined exercise programs, are more effective for reducing BM and SFA than resistance exercise in patients with T2DM. However, aerobic exercise as a standalone type of exercise training is more effective for reducing liver fat than combined exercise. In addition, both aerobic and combined training were more effective for decreasing VFA than resistance training, as compared to a control. However, additional data are required to clearly evaluate the efficacy of interventions that involve resistance training on hepatic fat. It is noteworthy that exercise training is effective in reducing VFA in T2DM patients with overweight and obesity. A key finding of this study is that the beneficial effect of exercise on VFA and SFA reduction (but not liver fat) is related to BM loss. These findings underscore the importance of including sustained exercise as a key component in the management of T2DM and its associated complications, with potential long-term benefits for metabolic health.

## Figures and Tables

**Table 1:** Participant and intervention characteristics.

Study	Sample Size (Sex)	Health Condition	Groups	Outcomes	Age [Years] Mean ± SD	BMI [kg/m^2^] Mean ± SD	Exercise Intervention	Follow Up (Week)	Diet Intervention
Abdelbassett et al. (2019) [30]	32 (M and F)	T2DM NAFLD Obesity	Con HIIT	VFA (cm^2^) Liver fat (%)	Con: ccc HIIT: 54.4 ± 5.8	Con: 35.9 ± 5.3 HIIT: 36.3 ± 4.5	5 min warm-up and 3 sets of 4 min cycling sessions at 80% to 85% of the VO2max with 2 min intervals at 50% of the VO2max between sets and 5 min cool-down × 3 d/w	8 weeks	Medical treatment Each patient was instructed to not eat for 2 h before the exercise session to avoid exercise-induced airway obstruction
Abdelbasset et al. (2020) [1]	42 (M and F)	T2DM NAFLD Obesity	Con HIIT MICT	VFA (cm^2^) Liver fat (%)	Con: 55.2 ± 4.3 HIIT: 54.4 ± 5.8 MICT: 54.9 ± 4.7	Con: 35.9 ± 5.3 HIIT: 36.3 ± 4.5 MICT: 36.7 ± 3.4	HIIT: 5 min warm-up and 3 sets of 4 min cycle Ergometer at 80% to 85% of the VO2max with 2 min interval at 50% of the VO2max between sets and 5 min cool-down × 3 d/w MICT: 5 min warming up followed by 40–50 min cycle ergometer with continuous intensity at 60% to 70% max HR and 5 min cooling down × 3 d/w	8 weeks	NR
Barone et al. (2012) [31]	112 (M and F)	T2DM Obesity	Con Combined Exe (A-Exe + R-Exe)	VFA (cm^2^)	Con: 56 ± 6 Combined Exe: 58 ± 5	Con: 33.5 ± 4.3 Combined Exe: 32.35.3	A-Exe: 60 min of 60–90% MHR × 3 d/w R-Exe: 2 sets of 12–15 reps at 50% 1RM of machine weights × 3 d/w	26 weeks	NR
Bonekamp et al. (2008) [32]	45 (M and F)	T2DM Obesity	Con Combined Exe (A-Exe + R-Exe)	Liver fat (%)	58	31.4	A-Exe: 45 min of 80% MHR R-Exe: lifting 7 weights at 2 sets of 12–15 reps at 50% 1RM × 3 d/w	26 weeks	NR
Botton et al. (2018) [33]	26 (M and F)	T2DM	Con R-Exe	VFA (mm)	Con: 68.6 ± 7.06 R-Exe: 70.6 ± 6.7	Con: 28.64 ± 3.26 R-Exe: 28.2 ± 3.6	R-Exe: whole-body exercise; 2–3 sets with 10–15 reps and 60–90 s rest between each set × 3 d/w	12 weeks	NR
Bouchi et al. (2021) [34]	141 (M and F)	T2DM	Con: DAPA Con R-Exe: DAPA + R-Exe	Trunk fat mass (kg) Body weight (kg)	Con: 57 ± 11 R-Exe: 59 ± 10	Con: 25.5 ± 4 R-Exe: 25.7 ± 3.5	30 min walking and resistance training of three sets of 10 repetitions of six items × daily	24 weeks	DAPA was administered from a starting dose of 5 mg to both groups, and participants were allowed to increase the dose up to 10 mg after ≥4 weeks if they failed to achieve the target HbA1c of <7.0%
Boudou et al. (2001) [23]	16 (M)	T2DM	Con A-Exe (A-Exe1 + A-Exe2)	VFA (cm^2^) SAT (cm^2^) Body weight (kg)	Con: 45.4 ± 7.2 A-Exe: 45.4 ± 7.2	Con: 29.6 ± 4.6 A-Exe: 29.6 ± 4.6	A-Exe1: 40 min of continuous cycle ergometer exercise was performed at 75% VO2peak × 2 d/w A-Exe2: cycle ergometer exercise was performed 20 min with 5 × 2 min 85% VO2peak work periods and 3 min 50% VO2peak rest periods × 1 d/w	8 weeks	NR
Bozzetto et al. (2012) [35]	17 (M and F)	T2DM Obesity	Con1: MUFA diet Con A-Exe1: MUFA diet + A-Exe Con2: CHO/fiber Diet A-Exe2: CHO/fiber Diet + A-Exe	Liver fat (%) Body weight (kg)	Con1: 57 ± 8 A-Exe1: 57 ± 9 Con2: 58 ± 5 A-Exe2: 57 ± 9	Con1: 28 ± 3 A-Exe1: 30 ± 4 Con2: 30 ± 2 A-Exe2: 31 ± 3	A-Exe: 45 min treadmill or cycle ergometer at 70% baseline VO2peak + warm-up and cool-down × 2 d/w	8 weeks	High-MUFA diet for both groups enforced by calls from dietician every 2–3 days
Cassidy et al. (2016) [36]	23 (M and F)	T2DM Obesity	Con HIIT	VFA (cm^2^) Liver fat (%) Body weight (kg)	Con: 59 ± 9 HIIT: 61 ± 9	Con: 32 ± 6 HIIT: 31 ± 5	HIIT: cycle ergometer and passive recovery at RPE 9–13 during warm-up and 5 intervals at an RPE 16–17 during high-intensity interval. Interval duration started at 2 min and progressed to 3 min and 50 s by week 12 × 3 d/w	12 weeks	NR
Celli et al. (2022) [26]	100 (M and F)	T2DM Obesity	Con Combined Exe (A-Exe + R-Exe)	VFA (cm^3^) Body weight (kg)	Con: 71.4 ± 3.7 Combined Exe: 72.3 ± 4.01	Con: 34.5 ± 5.4 Combined Exe: 35.7 ± 5.1	15 min warm-up flexibility exercises followed by, 30 min aerobic exercises (65–85% PHR), 30 min resistance exercises (1–2 sets, 8–12 repetitions at 65–85% of 1RM), and 15 min balance exercises	52 weeks	calcium and vitamin D intaketo 1500 mg/day and 1000 IU/day, respectively
Choi et al. (2012) [48]	75 (F)	T2DM	Con MICT	VFA (cm^2^) SAT (cm^2^) Body weight (kg)	Con: 55 ± 6.0 MICT: 53.8 ± 7.2	26.8 ± 2.4	MICT: 60 min walking of 3.6–6.0 METs × 5 d/w	12 weeks	NR
Cuff et al. (2003) [49]	28 (F)	T2DM Obesity Postmenopausal	Con A-Exe Combined Exe (A-Exe + R-Exe)	VFA (cm^2^) IMCL (cm^2^) SAT (cm^2^) Body weight (kg)	Con: 60 ± 7.9 A-Exe: 63.4 ± 6.9 Combined Exe: 59.4 ± 5.7	Con: 36.7 ± 6.0 A-Exe: 33.3 ± 4.7 Combined Exe: 32.5 ± 4.2	A-Exe: 75 min of treadmill, cycle ergometers, recumbent steppers and elliptical trainers at 60–75% HRR R-Exe: 5 exercises of stack weight equipment. 2 sets of 12 reps A-Exe + R-Exe 75 min × 3 d/w	16 weeks	NR
Dobrosielski et al. (2012) [37]	140 (M and F)	T2DM Obesity	Con Combined Exe (A-Exe + R-Exe)	VFA (cm^2^) SAT (cm^2^) Body weight (kg)	Con: 56 ± 6 Combined Exe: 57 ± 6	Con: 33.6 ± 0.5 Combined Exe: 33.0 ± 0.6	A-Exe = 45 min of treadmill, stationary cycle, or stair stepper at 60–90% MHR R-Exe = multistation machine 2 sets of 10–15 reps at 50% 1RM × 3 d/w	26 weeks	All participants were given dietary advice from the American Heart Association
Giannopoulou et al. (2005) [50]	33 (F)	T2DM Obesity Postmenopausal	Con: HMF Diet HMF Diet + A-Exe A-Exe	VFA (cm^3^) SAT (cm^3^) Body weight (kg)	Con: 58.5 ± 1.7 Diet + A-Exe: 57.5 ± 1.7 A-Exe: 55.5 ± 1.7	Con: 34.3 ± 1.9 Diet + A-Exe: 33.7 ± 1.9 A-Exe: 35.9 ± 1.9	A-Exe: walking at 60–70% VO2peak × 3–4 d/w	10 weeks	High-monounsaturated-fat diet composed of 40% fat (30% monounsaturated, 5% polyunsaturated, and 5% saturated), 40% carbohydrates (15% simple and 25% complex carbohydrates), and 20% protein = ~ 2510 kJ/day on non-exercise days and ~1460 kJ deficit where applicable
Jiang et al. (2020) [38]	49 (M and F)	T2DM	Con (M) Con (F) A-Exe (M) A-Exe (F)	VFA (cm^2^) SAT (cm^2^) Body weight (kg)	Con (M): 62.6 ± 3.8 Con (F): 62.6 ± 3.8 A-Exe (M): 63.9 ± 6.1 A-Exe (F): 63.9 ± 6.1	Con (M): 26.5 ± 2.1 Con (F): 26.7 ± 3.2 A-Exe (M): 26.9 ± 2.1 A-Exe (F): 26.6 ± 2.2	30–60 min walking/running at FATmax HR × 3 d/w	16 weeks	All participants were required to record a five-weekday dietary diary at the beginning and the end of the experimental period. The weight of the food and percentages of carbohydrate, fat, and protein in the food were estimated from the records
Jung et al. (2012) [51]	28 (F)	T2DM	Con A-Exe1 A-Exe2	VFA (cm^2^) SAT (cm^2^) Body weight (kg)	Con: 55.5 ± 7.6 A-Exe1: 56.8 ± 8.2 A-Exe2: 48.4 ± 6.1	Con: 27.7 ± 3.4 A-Exe1: 25.5 ± 1.5 A-Exe2: 25.9 ± 1.6	MICT: 60 min moderate intensity walking exercise at 3.5–5.2 METs A-Exe: 30 min vigorous intensity walking exercise at >5.3 METs × 5 d/w	12 weeks	NR
Jung et al. (2014) [52]	35 (F)	T2DM	Con A-Exe	VFA (cm^2^) SAT (cm^2^) Body weight (kg)	Con: 57.6 ± 3.5 A-Exe: 55.4 ± 3.5	Con: 27.2 ± 2.1 A-Exe: 26.0 ± 1.5	A-Exe: 60 min of walking exercise at 3.6–5.2 METs × 3 d/w	12 weeks	Both groups received one dietary education program at the beginning of the intervention
Karstoft et al. (2013) [39]	32 (M and F)	T2DM	Con A-Exe1 A-Exe2	VFA (L) Body weight (kg)	Con: 57.1 ± 3.0 A-Exe1: 60.8 ± 2.2 A-Exe2: 57.5 ± 2.4	Con: 29.7 ± 1.9 A-Exe1: 29.9 ± 1.6 A-Exe2: 29.0 ± 1.3	A-Exe1: 60 min of interval walking exercise 3 min at 70% of the peak energy expenditure rate during intense interval and 3 min at A-Exe2: 60 min of Continuous walking exercise 55% of the peak energy expenditure rate × 5 d/w	16 weeks	NR
Kong et al. (2022) [40]	86 (M and F)	T2DM	Con A-Exe	VFA (cm^2^) Body weight (kg)	Con: 50 ± 8 A-Exe: 50 ± 10	Con: 29 ± 4 A-Exe: 28 ± 5	Low intensity to 70% of maximum heart rate- 60–90 min each time × 3–5 d/w	16 weeks	1 week before the experiment, fat, rice or noodleswere minimized to about 250 g per day
Koo et al. (2010) [53]	64 (F)	T2DM	Con1 A-Exe1 Con2: Diet A-Exe2: Diet + A-Exe	VFA (cm^2^) SAT (cm^2^) Body weight (kg)	Con1: 57 ± 8 A-Exe1: 59 ± 4 Con2: 57 ± 8 A-Exe2: 53 ± 8	Con1: 28.5 A-Exe1: 25.5 Con2: 27.1 A-Exe2: 29.4	A-Exe and Diet + A-Exe: 120 min brisk walking × 7 d/w	12 weeks	C and A-Exe: received conventional education for a mildly hypocaloric diet (30 kcal per kg of ideal body weight per day) at the beginning of the study Diet and Diet + A-Exe: reduced their usual energy intake to 1200 kcal/day for weight reduction and were educated individually every 2 weeks based on the self-recorded 3-day diet diary
Ku et al. (2010) [54]	44 (F)	T2DM	Con R-Exe A-Exe	VFA (g) IMCL (g) SAT (cm^2^) Body weight (kg)	Con: 57.8 ± 8.1 R-Exe: 55.7 ± 6.2 A-Exe: 55.7 ± 7.0	Con: 27.4 ± 2.8 R-Exe: 27.1 ± 2.3 A-Exe: 27.1 ± 2.4	R-Exe: 3 sets of 15–20 repetitions at 40–50% 1RM A-Exe: 60 min walking at 3.6–5.2 METs × 5 d/w	12 weeks	NR
Kwon et al. (2010) a [55]	28 (F)	T2DM	Con R-Exe	VFA (mm^2^) SAT (mm^2^) Body weight (kg)	Con: 57.0 ± 8.0 R-Exe: 55.7 ± 6.2	Con: 27.6 ± 2.8 R-Exe: 27.1 ± 2.3	R-Exe: 40 min 3 sets of 10–15 reps at 40–50%1RM and 20 min collectively of warm-up and cool-down × 3 d/w	12 weeks	Three-day diet record (two weekdays and one weekend day) and visited the hospital every four weeks to have their dietary record reviewed
Kwon et al. (2010) b [56]	27 (F)	T2DM	Con A-Exe	VFA (mm^2^) SAT (mm^2^)	Con: 57.5 ± 8.6 A-Exe: 55.5 ± 7.5	Con: 27.5 ± 3.0 A-Exe: 27.0 ± 2.5	A-Exe: performed 60 min moderate intensity walking × 5 d/w	12 weeks	Three-day diet record (two weekdays and one weekend day) and visited the clinic every four weeks to have their dietary record reviewed
Li et al. (2022) [27]	82 (M and F)	T2DM	Con A-Exe	VFA (cm^2^) SAT (cm^2^)	Con: 67.62 ±5.91 A-Exe: 65.15 ± 5.00	Con: 24.77 ± 3.02 A-Exe: 24.27 ± 2.76	A-Exe: 5 min warm-up, 50 minaerobic dancing, 5 min cool-down (60%–70% ofMHR) × 3 d/w	24 weeks	All participants follow ahealthy diet (55–60% carbohydrate, 15–20% protein, and 25–30% fat
Lyngbæk et al. (2023) [41]	44 (M and F)	T2DM Obesity	Con: Diet Diet + Combined Exe1 Diet + Combined Exe2	VFA (cm^3^) SAT (cm^3^) Body weight (kg)	Con: 55.9 ± 10.0 Combined Exe1: 60.9 ± 7.6 Combined Exe2: 57.3 ± 11.8	Con: 33.2 ± 3.8 Combined Exe1: 33.2 ± 4.1 Combined Exe2: 33.4 ± 3.5	Combined Exe1: two aerobic training sessions and one combined aerobic andresistance training session/per week in total of 150–165 min. Combined Exe2: four aerobic training sessions/per week and two sessions/per week with combined aerobic in total of 300–330 min. training and resistance training. 60–100% HRmax. 8–12 repetitions.	16 weeks	DI: ~25–30% energy deficit/day (45–60E% carbohydrate, 15–20E% protein, and 20–35E% fat (<7E% saturatedfat).
Mavros et al. (2013) [42]	83 (M and F)	T2DM Obesity	Con R-Exe	VFA (cm^2^) IMCL (cm^2^) Body weight (kg)	Con: 68.9 ± 6.0 R-Exe: 67.1 ± 4.8	Con: 31.5 ± 6.3 R-Exe: 31.0 ± 4.6	R-Exe: 80% 1RM power training, quick concentric phase and slow eccentric phase × 3 d/w	52 weeks	NR
Mourier et al. (1997) [25]	21 (M and F)	T2DM Obesity	Con A-Exe	VFA (cm^2^) SAT (cm^2^) Body weight (kg)	Con: 46 ± 9.9 A-Exe: 45 ± 6.3	Con: 30.1 ± 5.3 A-Exe: 30.4 ± 2.5	A-Exe: 55 min continuous cycling at 75% VO2peak; ×2 d/w, 35 min intermittent exercise was performed 5×2 min at 85% VO2peak and 3 min of 50% VO2peak recovery periods× 1 d/w	12 weeks	Half of the participants from both groups were given BCAA capsules (46% leucine, 24% isoleucine, and 30% valine)
Otten et al. (2018) [43]	26 (M and F)	T2DM Obesity	Con: Paleolithic diet Combined Exe: Paleolithic diet + Exe	Liver fat (%) IMCL (%) Body weight (kg)	Con: 59.33 ± 9.30 Combined Exe: 62 ± 7.47	Con: 31.5 ± 3.57 Combined Exe: 31.6 ± 4.65	Aerobic exercise and resistance training in 60 min sessions × 3 d/w	12 weeks	The Paleolithic diet included lean meat, eggs, fish, seafood, nuts, fruits and vegetables. Dairy products, cereals, legumes and added sugar and salt were excluded. Energy intake was ad libitum
Sabag et al. (2020) [28]	35 (M and F)	T2DM Obesity	Con MICT HIIT	Liver fat (%) Body weight (kg)	Con: 54.8 ± 8.3 MICT: 56.9 ± 7.2 HIIT: 51.9 ± 4.6	Con: 35.8 ± 5.6 MICT: 34.3 ± 3.8 HIIT: 37.5 ± 5.5	MICT: 30–55 min of Continuous cycling at 60% VO2peak × 3 d/w HIIT: 1–4 min of cycling at a 90% VO2peak and a 10 min warm-up and 5 min cool-down at a 50% VO2peak and 5 min warm-up and cool-down at 50% VO2peak × 3 d/w	12 weeks	NR
Sigal et al. (2007) [24]	251 (M and F)	T2DM Obesity	Con A-Exe R-Exe Combined Exe (A-Exe + R-Exe)	VFA (cm^2^) SAT (cm^2^) Body weight (kg)	Con: 54.8 ± 7.2 A-Exe: 53.9 ± 6.6 R-Exe: 54.7 ± 7.5 Combined Exe: 53.5 ± 7.3	Con: 35.0 ± 9.5 A-Exe: 35.6 ± 10.1 R-Exe: 34.1 ± 9.6 Combined Exe: 35.0 ± 9.6	A-Exe: 15–45 min treadmill or bicycle exercise at 60–75% MHR× 3 d/w R-Exe: 2–3 sets of 7–9 RM machine weights × 3 d/w A-EXE + PRT: completed the full exercise programs for A-Exe and PRT × 3 d/w	26 weeks	Standardized diet counseling given to all participants at the beginning of the trial based on the Canadian Diabetes Diet recommendations
Snel et al. (2012) [29]	27 (M and F)	T2DM Obesity	VLCD VLCD + A-Ex	IMCL (cm^2^) Body weight (kg)	Con: 56.1 ± 2.4 A-Exe: 53.0 ± 2.5	Con: 37.9 ± 1.4 A-Exe: 36.4 ± 1.1	AEx: 60 min cycle ergometer at 3.6–5.2 METs × 3 d/w	16 weeks	All patients started a 16 wk VLCD (Modifast, Nutrition & Sante, Antwerpen, Belgium). Modifast provides a total of approximately 450 kcal/d and all necessary vitamins and micronutrients, divided over three meals of liquid shakes
Stomby et al. (2020) [44]	28 (M and F)	T2DM Obesity	Con: Paleolithic diet Combined Exe: Paleolithic diet + Exe	Liver fat (%)	Con: 60 ± 11 Combined Exe: 61 ± 8	Con: 31.4 ± 4.3 Combined Exe: 31.4 ± 6.1	60 min aerobic and resistance exercise at 50% × 3 d/w	12 weeks	The Paleolithic-type diet included recommendations of a high intake of vegetables, fruit, lean meat, nuts, eggs, fish and seafood. The intake of grains, sugar, salt, dairy products and refined fats was reduced
Szilagyi et al. (2019) [45]	208 (M and F)	T2DM Obesity	Con Combined Exe (A-Exe + R-Exe)	VFA (cm^2^) Body weight (kg)	Con: 60.10 ± 7.32 Combined Exe: 61.83 ± 6.86	Con: 33.64 ± 4.31 Combined Exe: 33.63 ± 4.09	10 min warm-up and 40 min aerobic exercise MAX. Pulse 60–75% and 10 min resistance training and 10 min cool-down × 3–4 d/w	24 weeks	Exercise diary (concentration of glucose in blood pressure, pulse, ketone body) was kept regularly.
Tan et al. (2018) [57]	31 (F)	T2DM	Con A-Exe	Visceral trunk fat (%) Body weight (kg)	Con: 62.9 ± 2.6 A-Exe: 63.0 ± 2.3	Con: 26.5 ± 3.2 A-Exe: 26.6 ± 3.1	40–60 min at fat maxHR of walking or running × 3 d/w	12 weeks	Daily energy intake was then calculated by multiplying the proportions of carbohydrate, fat and protein consumed with their respective energy values (carbohydrate provides 4 kcal/g of energy, fat 9 kcal/g and protein 4 kcal/g)
Winding et al. (2018) [46]	32 (M and F)	T2DM	Con A-Exe HIIT	VFA (kg) Body weight (kg)	Con: 57 ± 7 A-Exe: 58 ± 8 HIIT: 54 ± 6	Con: 28.0 ± 3.5 A-Exe: 27.4 ± 3.1 HIIT: 28.1 ± 3.5	A-Exe: 5 min warm-up, 40 min of cycling at 50% of Wpeak × 3 d/w HIIT: 5 min warm-up, 20 min of cycling consisting of cycles of 1 min at 95% Wpeak and 1 min of active recovery (20% Wpeak) × 3 d/w	11 weeks	On experimental days, participants refrained from taking their anti-diabetic medication and arrived in a fasting state (≥10 h). Participants refrained from alcohol and caffeine intake for at least 24 h prior to any of the testing days and from exercise for 24 or 48 h before test days A and B, respectively
Yamaguchi et al. (2011) [47]	19 (M and F)	T2DM	Con A-Exe	VFA (cm^2^) SAT (cm^2^) Body weight (kg)	Con: 50 ± 2.7 A-Exe: 50 ± 3.1	Con: 27.8 ± 5.6 A-Exe: 27.9 ± 6.0	A-Exe: 2 × 30 min bouts each day of walking exercise at 3.6–5.2 METs × 7 d/w	12 weeks	Both groups received one dietary education program at the beginning of the intervention

Abbreviations: M: male; F: female; BMI: body mass index; mg: milligram; kcal: kilocalorie; kJ: kilojoule; min: minutes; T2DM: type 2 diabetes mellitus; RPE: rate of perceived exertion; Con: control; D: diet; HMF: high monounsaturated fat; SAT: subcutaneous adipose tissue; VFA: visceral fat area; AVFA: abdomen visceral adipose tissue; A-Exe: aerobic exercise; R-Exe: resistance exercise; Exe: exercise; HIIT: high-intensity interval training; MICT: moderate-intensity aerobic exercise; SIT: sprint interval training; PLA: placebo; PD: Paleolithic diet; VLCD: very-low-calorie diet; NR: not reported; VO2peak: peak rate of oxygen consumption; Wpeak: peak power output; HRR: heart rate reserve; HR: heart rate; MHR: maximum heart rate; METs: metabolic equivalent; AT: anaerobic threshold; RM: repetition maximum; CHO: carbohydrates; MUFAs: monounsaturated fatty acids; BCAAs: branched-chain amino acids; VO2peak: peak oxygen consumption; Kg: kilogram; g: gram; DAPA: dapagliflozin.

**Table 2:** Effects of exercise training vs. control on ectopic and subcutaneous fat.

Reference	Mode	Measure	Pre. Mean (±SD)	Post. Mean (±SD)	Mean Change
Abdelbassett et al. (2019) [30]	Con: *n* = 16 HIIT: *n* = 16	VFA: MRI Liver fat: MRI	VFA:	VFA:	
			Con: 179.8 ± 14.4	Con: 177.2 ± 12.8	NR
			HIIT: 184.5 ± 12.3	HIIT: 166.4 ± 11.6	NR
			Liver fat:	Liver fat:	
			Con: 11.2 ± 5.1	Con: 11.1 ± 5.2	NR
			HIIT: 12.4 ± 4.5	HIIT: 10.1 ± 1.3	NR
Abdelbasset et al. (2020) [1]	Con: *n* = 16 HIIT: *n* = 16 MICT: *n* = 15	VFA: MRI Liver fat: MRI	VFA:	VFA:	
			Con: 179.8 ± 14.4	Con: 177.2 ± 12.8	NR
			HIIT: 184.5 ± 12.3	HIIT: 166.4 ± 11.6	NR
			MICT: 181.7 ± 13.5	MICT: 170.3 ± 10.6	NR
			Liver fat:	Liver fat:	
			Con: 11.2 ± 5.1	Con: 11.1 ± 5.2	NR
			HIIT: 12.4 ± 4.5	HIIT: 10.1 ± 1.3	NR
			MICT: 12.9 ± 4.2	MICT: 10.5 ± 1.5	NR
Barone et al. (2012) [31]	Con: *n*= 63 Combined Exe (A-Exe + R-Exe): *n*= 49	VFA: MRI	VFA:	VFA:	
			Con: 169 ± 75	Con: 167 ± 71	Con: −2 ± 36
			Combined Exe: 155 ± 70	Combined Exe: 149 ± 68	Combined Exe: −6 ± 33
Bonekamp et al. (2008) [32]	Con: *n* = 20 Combined Exe (A-Exe + R-Exe): *n* = 25	Liver fat: H-MRS	Liver fat:	Liver fat:	
			Con: 7.45 ± 5.65	Con: 8.5 ± 6.09	NR
			Combined Exe: 6.8 ± 5.15	Combined Exe: 5.78 ± 4.93	NR
Botton et al. (2018) [33]	Con: *n* = 13 R-Exe: *n* = 13	VFA: Ultrasonography	VFA:	VFA:	
			Con: 73.10 ± 22.80	Con: 68.18 ± 21.04	NR
			R-Exe: 78.41 ± 17.38	7 R-Exe: 3.75 ± 17.57	NR
Bouchi et al. (2021) [34]	Con: *n* = 69 R-Exe: *n* = 72	Trunk fat mass: DXA	Trunk fat mass:	Trunk fat mass:	
			Con: 12.3 ± 5.2	Con: 11.5 ± 4.9	Con: −0.9 ± 1.2
			R-Exe: 12.9 ± 5.1	R-Exe: 11.5 ± 5.1	R-Exe: −1.5 ± 1.2
Boudou et al. (2001) [23]	Con: *n* = 8 A-Exe: *n* = 8	VFA: MRI SAT: MRI	VFA:	VFA:	
			Con: 156.85 ± 23.40	Con: 150.35 ± 23.25	NR
			A-Exe: 153.25 ± 38.55	A-Exe: 84.20 ± 21.30	NR
			SAT:	SAT:	
			Con: 262.50± 69.10	Con: 260.00± 70.40	NR
			A-Exe: 241.55± 49.55	A-Exe: 198.00 ± 39.00	NR
Bozzetto et al. (2012) [35]	MUFA diet: *n* = 8 MUFA diet + A-Exe: *n* = 9 CHO Diet: *n* = 9 CHO Diet + A-Exe: *n* = 10	Liver fat: H-MRS	Liver fat:	Liver fat:	
			MUFA diet: 7.4 ± 2.8	MUFA diet: 5.2 ± 2.7	NR
			A-Exe1: 11.6 ± 8.0	A-Exe1: 9.1 ± 7.0	NR
			CHO Diet: 17.7 ± 9.7	CHO Diet: 16.1 ± 6.8	NR
			A-Exe2: 8.8 ± 4.9	A-Exe2: 8.9 ± 5.7	NR
Cassidy et al. (2016) [36]	Con: *n* = 11 HIIT: *n* = 12	VFA: MRI Liver fat: H-MRS	VFA:	VFA:	
			Con: 159 ± 58	Con: 181 ± 72	NR
			HIIT: 201 ± 80	HIIT: 146.8 ± 36.7	NR
			Liver fat:	Liver fat:	
			Con: 7.1 ± 6.8	Con: 7.7 ± 6.9	NR
			HIIT: 6.9 ± 6.9	HIIT: 4.2 ± 3.6	NR
Celli et al. (2022) [26]	Con: *n* = 50 Combined Exe (A-Exe + R-Exe): *n* = 50	VFA: DEXA	VFA:		VFA:
			Con: 1196 ± 403	NR	Con: −30 ± 190.9
			Combined Exe: 1203 ± 353.5	NR	Combined Exe: −261 ± 205
Choi et al. (2012) [48]	Con: *n* = 37 MICT: *n* = 38	VFA: CT scan SAT: CT scan	VFA:	VFA:	
			Con: 149.2 ± 41.5	Con: 146.8 ± 36.7	NR
			MICT: 153.7 ± 38.9	MICT: 136.6 ± 39.2	NR
			SAT:	SAT:	
			Con: 217.6 ± 58.1	Con: 217.6 ± 58.1	NR
			MICT: 219.7 ± 64.4	MICT: 200.3 ± 59.7	NR
Cuff et al. (2003) [49]	Con: *n* = 9 A-Exe: *n* = 9 Combined Exe (A-Exe + R-Exe): *n* = 10	VFA: CT/i scanner IMCL: CT/i scanner SAT: CT/i scanner	VFA:		VFA:
			Con: 259.1 ± 103.2	NR	Con: −0.4 ± 36
			A-Exe: 215.7 ± 77.4	NR	A-Exe: −8.8 ± 16.2
			Combined Exe: 251.1 ± 72.4	NR	Combined Exe: −26.3 ± 23.4
			IMCL:		IMCL:
			Con: 225.8 ± 26.7	NR	Con: 0.7 ± 4.8
			A-Exe: 224.1 ± 47.4	NR	A-Exe: 0.9 ± 6.3
			Combined Exe: 208 ± 3162	NR	Combined Exe: 5.9 ± 6.3
			SAT:		SAT:
			Con: 549.3 ± 152.4	NR	Con: 17.4 ±27
			A-Exe: 401.2 ± 128.7	NR	A-Exe: −8.2 ± 29.1
			Combined Exe: 468.9 ± 85.06	NR	Combined Exe: −22.0 ± 48.69
Dobrosielski et al. (2012) [37]	Con: *n* = 63 Combined Exe (A-Exe + R-Exe): *n* = 51	VFA: MRI SAT: MRI	VFA:	VFA:	VFA:
			Con: 165.2 ± 74.4	Con: 161.3 ± 70.6	Con: −2.1 ± 36.51
			Combined Exe: 153.3 ± 68.6	Combined Exe: 145.0 ± 60.7	Combined Exe: −8.1 ± 37.13
			SAT:	SAT:	SAT:
			Con: 404.7 ± 119.85	Con: 401.4 ± 126.99	Con: −6.1 ± 12.69
			Combined Exe: 399.9 ± 122.11	Combined Exe: 381.6 ± 114.26	Combined Exe: −20.0 ± 12.14
Giannopoulou et al. (2005) [50]	Diet: *n* = 11 Diet + A-Exe: *n* = 11	VFA: MRI SAT: MRI	VFA:	VFA:	
			Con: 4785 ± 1592	Con: 4425 ± 1442.7	NR
			A-Exe: 5912 ± 1605.2	A-Exe: 5152 ± 1456	NR
			SAT:	SAT:	
			Con: 9900.85 ± 3100	Con: 9093.48 ± 3382.3	NR
			A-Exe: 10,028.3 ± 3100	A-Exe: 8966.01 ± 3100.5	NR
Jiang et al. (2020) [38]	Con: *n*: M = 11 F = 13 A-Exe: *n*: M = 14 F = 11	VFA: bioelectrical impedance analysis equipment SAT: bioelectrical impedance analysis equipment	VFA:	VFA:	
			Con (M): 103.9 ± 44.8	Con (M): 112.4 ± 47.1	NR
			Con (F): 88.2 ± 23.8	Con (F): 91.5 ± 21.0	NR
			A-Exe (M): 106.2 ± 33.5	A-Exe (M): 91.8 ± 29.6	NR
			A-Exe (F): 85.6 ± 40.2	A-Exe (F): 75.0 ± 27.6	NR
			SAT:	SAT:	
			Con (M): 213.7 ± 69.4	Con (M): 220.5 ± 68.1	NR
			Con (F): 212.5 ± 46.4	Con (F): 220.8 ± 49.1	NR
			A-Exe (M): 214.4 ± 39.7	A-Exe(M): 192.7 ± 40.6	NR
			A-Exe (F): 209.9 ± 58.3	A-Exe (F): 192.7 ± 53.7	NR
Jung et al. (2012) [51]	Con: *n* = 12 A-Exe1: *n* = 8 A-Exe2: *n* = 8	VFA: visceral fat cm2 via CT SAT: CT	VFA:	VFA:	
			Con: 17,790.2 ± 5621.7	Con: 17,372.7 ± 5235.7	NR
			A-Exe1: 15,784.6 ± 4662.7	A-Exe1: 13,262.5 ± 3217.8	NR
			A-Exe2: 13,726.6 ± 3011.8	A-Exe2: 12,447.4 ± 2252.6	NR
			SAT:	SAT:	
			Con: 22,153.9 ± 5700.5	Con: 22,627.6 ± 5799.0	NR
			A-Exe1: 19,413.1 ± 3265.9	A-Exe1: 18,441.1 ± 3215.2	NR
			A-Exe2: 18,669.4 ± 5027.3	A-Exe2: 17,311.1 ± 5306.8	NR
Jung et al. (2014) [52]	Con: *n* = 18 A-Exe: *n* = 17	VFA: visceral fat cm2 via CT SAT: CT	VFA:	VFA:	
			Con: 16,175.2 ± 4296.6	Con: 16,175.2 ± 4196.6	NR
			A-Exe: 14,757.1 ± 2708.5	A-Exe: 14,757.1 ± 2708.5	NR
			SAT:	SAT:	
			Con: 23,186.1 ± 5858.8	Con: 21,957.2 ± 5563.5	NR
			A-Exe: 20,333.0 ± 6297.7	A-Exe: 18,217.4 ± 6310.8	NR
Karstoft et al. (2013) [39]	Con: *n* = 8 MICT: *n* = 12 HIIT: *n* = 12	VFA: MRI	VFA:	VFA:	
			Con: 4.7 ± 1.1	Con: 4.6 ± 1.3	NR
			4.5 ± 1.0	4.2 ± 1.3	NR
			4.7 ± 2.7	4.2 ± 2.4	NR
Kong et al. (2022) [40]	Con: *n* = 43 A-Exe: *n* = 43	VFA: CT scanner	VFA:	VFA:	
			Con: 118.1 ± 33.7	Con: 118.8 ± 32.5	NR
			117.5 ± 32.7	94.2 ± 31.7	NR
Koo et al. (2010) [53]	Con: *n* = 18 A-Exe: *n* = 13 Diet: *n* = 19 Diet + A-Exe: *n* = 14	VFA: CT SAT: CT	VFA:	VFA:	VFA:
			Con1: 172.4	Con1: 163.4	Con1: −8.0 ± 30.3
			A-Exe1: 162.4	A-Exe1: 146.9	A-Exe1: −29.7 ± 23.3
			Con2: 157.8	Con2: 151.7	Con2: −19.5 ± 28.0
			A-Exe2: 152.7	A-Exe2: 120.0	A-Exe2: −38.2 ± 26.0
			SAT:	SAT:	SAT:
			Con1: 208.1	Con1: 204.0	Con1: 0.1 ± 21.4
			A-Exe1: 219.0	A-Exe1: 220.0	A-Exe1: −16.8 ± 23.6
			Con2: 216.5	Con2: 196.1	Con2: −27.6 ± 27
			A-Exe2: 263.9	A-Exe2: 231.7	A-Exe2: −26.5 ± 25.2
Ku et al. (2010) [54]	Con: *n* = 16 R-Exe: *n* = 13 A-Exe: *n* = 15	VFA: CT (g) IMCL: CT SAT: CT	VFA:	VFA:	VFA:
			Con: 17,530 ± 4747	Con: 17,362 ± 4728	Con: −168 ± 1801
			R-Exe: 15,658 ± 4754	R-Exe: 14,678 ± 3456	R-Exe: −980 ± 2353
			A-Exe: 15,890 ± 4593	A-Exe: 15,038 ± 3369	A-Exe: −852 ± 2839
			IMCL:	IMCL:	IMCL:
			Con: 564 ± 222	Con: 532 ± 215	Con: −32 ± 171
			R-Exe: 412 ± 160	R-Exe: 416 ± 159	R-Exe: 4 ± 199
			A-Exe: 509 ± 178	478 ± 184	A-Exe: −31 ± 159
			SAT:	SAT:	SAT:
			Con: 7371 ± 2620	Con: 7313 ± 2479	Con: −58 ± 1316
			R-Exe: 6697 ± 2674	R-Exe: 7660 ± 2760	R-Exe: 963 ± 1157
			A-Exe: 7187 ± 2960	A-Exe: 7849 ± 2510	A-Exe: 662 ± 966
Kwon et al. (2010) a [55]	Con: *n* = 15 R-Exe: *n* = 13	VFA: CT SAT: CT	VFA:	VFA:	
			Con: 17,268.7 ± 5060.9	Con: 17,745.1 ± 4715.3	NR
			R-Exe: 15,657.8 ± 4753.6	R-Exe: 14,677.8 ± 3455.9	NR
			SAT:	SAT:	
			Con: 24,357.5 ± 5437.8	Con: 23,721.7 ± 5131.6	NR
			R-Exe: 24,402.4 ± 7903.2	R-Exe: 22,731.9 ± 7264.2	NR
Kwon et al. (2010) b [56]	Con: *n* = 14 A-Exe: *n* = 13	VFA: CT SAT: CT	VFA:	VFA:	
			Con: 17,204.5 ± 4674.4	Con: 17,216.3 ± 4560.8	NR
			A-Exe: 16,291.5 ± 4808.5	A-Exe: 14,682.7 ± 3494.7	NR
			SAT:	SAT:	
			Con: 25,152.9 ± 5839.3	Con: 24,664.6 ± 5580.4	NR
			A-Exe: 23,891.9 ± 6439.1	A-Exe: 21,803.7 ± 6153.7	NR
Li et al. (2022) [27]	Con: *n* = 53 A-Exe: *n* = 53	VFA: MRI SAT: MRI	VFA:	VFA:	VFA:
			Con: 114.31 ± 56.60	Con: 117.90 ± 44.26	Con: 3.60 ± 19.26
			A-Exe: 104.37 ± 43.90	A-Exe: 96.24 ± 36.39	A-Exe: −8.13 ± 21.51
			SAT:	SAT:	SAT:
			Con: 177.86 ± 78.45	Con: 180.18 ± 76.80	Con: 2.31 ± 8.76
			A-Exe: 178.99 ± 66.66	A-Exe: 168.81 ± 64.09	A-Exe: −10.18 ± 22.43
Lyngbæk et al. (2023) [41]	Diet: *n* = 20 Diet + Combined Exe1: *n* = 20 Diet + Combined Exe2: *n* = 21	VFA: MRI SAT: MRI	VFA:		VFA:
			Con: 4659.5 ± 1719.8	NR	Con: −16.39 ± 5.83
			Combined Exe1: 4937.0 ± 1220.4	NR	Combined Exe1: −30.22 ± 16.3
			Combined Exe2: 4257.9 ± 1006.8	NR	Combined Exe2: −42.35 ± 11.66
			SAT:		SAT:
			Con: 4660.2 ± 686.3	NR	Con: −12.33 ± 7.8
			Combined Exe1: 3414.1 ± 504.2	NR	Combined Exe1: −18.69 ± 11
			Combined Exe2: 4127.6 ± 1341	NR	Combined Exe2: −29.97 ± 17.35
Mavros et al. (2013) [42]	Con: *n* = 46 R-Exe: *n* = 37	VFA: CT IMCL: CT	VFA:	VFA:	
			Con: 211.3 ± 93.9	Con: 210.7 ± 95.9	NR
			R-Exe: 215.0 ± 81.8	R-Exe: 202.3 ± 73.3	NR
			IMCL:	IMCL:	
			Con: 109.8 ± 21.7	Con: 109.4 ± 22.4	NR
			R-Exe: 112.5 ± 25.8	R-Exe: 117.8 ± 30.1	NR
Mourier et al. (1997) [25]	Con: *n* = 11 A-Exe: *n* = 10	VFA: MRI SAT: MRI	VFA:	VFA:	
			Con: 139.4 ± 36.8	Con: 134.9 ± 33.8	NR
			A-Exe: 156.1 ± 47.4	A-Exe: 80.4 ± 22.1	NR
			SAT:	SAT:	
			Con: 269.6 ± 64.34	Con: 260.3 ± 71.63	NR
			A-Exe: 227.3 ± 57.23	A-Exe: 186.7 ± 44.58	NR
Otten et al. (2018) [43]	Diet: *n* = 13 Diet + Combined Exe: *n* = 13	Liver fat: H-MRS IMCL: H-MRS	Liver fat:	Liver fat:	
			Con: 20.30 ± 8.48	Con: 11.713 ± 6.41	NR
			Combined Exe: 22.86 ± 15.47	Combined Exe: 19.32 ± 16.41	NR
			IMCL:	IMCL:	
			Con: 20.3 ± 8.48	Con: 11.71 ± 6.41	NR
			Combined Exe: 22.86 ± 15.47	Combined Exe: 19.32 ± 16.41	NR
Sabag et al. (2020) [28]	Con: *n* = 11 MICT: *n* = 12 HIIT: *n* = 12	Liver fat: H-MRS	Liver fat:	Liver fat:	
			Con: 11.8 ± 7.6	Con: 13.0 ± 8.9	NR
			MICT: 9.4 ± 6.9	MICT: 8.6 ± 7.2	NR
			HIIT: 9.7 ± 8.3	HIIT: 8.0 ± 7.6	NR
Sigal et al. (2007) [24]	Con: *n* = 63 A-Exe: *n* = 60 R-Exe: *n* = 64 Combined Exe (A-Exe + R-Exe): *n* = 64	VFA: CT SAT: CT	VFA:	VFA:	
			Con: 252 ± 147	Con: 250 ± 147	NR
			A-Exe: 257 ± 161	A-Exe: 244 ± 161	NR
			R-Exe: 228 ± 156	R-Exe: 218 ± 156	NR
			Combined Exe: 246 ± 159	Combined Exe: 224 ± 159	NR
			SAT:	SAT:	
			Con: 420 ± 209	Con: 416 ± 209	NR
			A-Exe: 448 ± 230	A-Exe: 431 ± 230	NR
			R-Exe: 412 ± 227	R-Exe: 394 ± 227	NR
			Combined Exe: 416 ± 230	Combined Exe: 389 ± 230	NR
Snel et al. (2012) [29]	Diet: *n* = 14 Diet + A-Exe: *n* = 13	IMCL: muscle biopsy	IMCL:	IMCL:	
			Con: 2 ± 1.5	Con: 1.28 ± 1.0	NR
			A-Exe: 1.88 ± 1.1	A-Exe: 1.16 ± 0.7	NR
Stomby et al. (2020) [44]	Diet: *n* = 15 Diet + Exe: *n* = 13	Liver fat: MRS	Liver fat:	Liver fat:	
			Con: 22 ± 20	Con: 5 ± 11	NR
			Exe: 14 ± 19	Exe: 10 ± 22	NR
Szilagyi et al. (2019) [45]	Con: *n* = 105 Combined Exe (A-Exe + R-Exe): *n* = 103	VFA: Omron Body Composition Monitor BF511	VFA:	VFA:	
			Con: 15.20 ± 5.55	Con: 16.52 ± 4.73	NR
			Combined Exe: 15.13 ± 5.85	Combined Exe: 14.76 ± 5.26	NR
Tan et al. (2018) [57]	Con: *n* = 15 A-Exe: *n* = 16	Visceral trunk fat: bioelectrical impedance analysis equipment	Visceral trunk fat:	Visceral trunk fat:	
			Con: 39.9 ± 7.5	Con: 40.1 ± 7.8	NR
			A-Exe: 39.6 ± 7.1	A-Exe: 34.7 ± 6.5	NR
Winding et al. (2018) [46]	Con: *n* = 7 A-Exe: *n* = 12 HIIT: *n* = 13	VFA: dual-energy X-ray absorptiometry	VFA:	VFA:	
			Con: 2.0 ± 0.6	Con: 2.1 ± 0.6	NR
			A-Exe: 1.6 ± 0.8	A-Exe: 1.4 ± 0.7	NR
			HIIT: 1.7 ± 0.8	HIIT: 1.5 ± 0.7	NR
Yamaguchi et al. (2011) [47]	Con: *n* = 8 A-Exe: *n* = 11	VFA: CT scan SAT: CT scan	VFA:	VFA:	
			Con: 162.8 ± 61.9	Con: 153.6 ± 55.8	NR
			A-Exe: 138.2 ± 56.5	A-Exe: 108.1 ± 51.6	NR
			SAT:	SAT:	
			Con: 193.1 ± 143.3	Con: 184.1 ± 145.6	NR
			A-Exe: 255.4 ± 139.1	A-Exe: 245.4 ± 132.9	NR

Abbreviations: *n*: number of subjects; Con: control; A-Exe: aerobic exercise; R-Exe: resistance exercise; D: diet; Exe: exercise; HIIT: high-intensity interval training; MICT: moderate-intensity aerobic exercise; SIT: sprint interval training; NR: no report; H-MRS: proton magnetic resonance spectroscopy; CT: computed tomography; MRI: magnetic resonance imaging; LA: liver attenuation; IMCL: intramyocellular lipid.

## Data Availability

All data and material reported in this systematic review are from peer reviewed publications.

## Supplementary Materials

The following supporting information can be downloaded at: https://www.mdpi.com/article/10.3390/jcm13175005/s1, PRISMA 2020 Checklist; Table S1: Risk of bias assessment (PEDro scale); Table S2: Search strategy. References [1,23,24,25,26,27,28,29,30,31,32,33,34,35,36,37,38,39,40,41,42,43,44,45,46,47,48,49,50,51,52,53,54,55,56,57,94] are cited in Supplementary Materials.

## Abbreviations

**M:** male; **F:** female; **BMI:** body mass index; **mg:** milligram; **kcal:** kilocalorie; **kj:** kilojoule; **min:** minutes; **T2DM:** type 2 diabetes mellitus; **RPE:** rate of perceived exertion; **Con:** control; **D:** diet; **HMF:** high monounsaturated fat; **SAT:** subcutaneous adipose tissue; **VFA:** visceral fat area; **AVFA:** abdomen visceral adipose tissue; **SFA:** subcutaneous fat area; **IMF:** intramuscular fat volume; **LA:** liver attenuation; **IMCL:** intramyocellular lipid; **A-Exe:** aerobic exercise; **R-Exe:** resistance exercise; **Exe:** exercise training; **PRE:** progressive resistance exercise; **HIIT:** high-intensity interval training; **MICT:** moderate-intensity aerobic exercise; **SIT:** sprint interval training; **PLA:** placebo; **PD:** Paleolithic diet; **VLCD:** very-low-calorie diet; **NR:** not reported; **VO2peak:** peak rate of oxygen consumption; **Wpeak:** peak power output; **HRR:** heart rate reserve; **HR:** heart rate; **MHR:** maximum heart rate; **METs:** metabolic equivalent; **AT:** anaerobic threshold; **RM:** repetition maximum; **CHO:** carbohydrates; **MUFAs:** monounsaturated fatty acids; **BCAAs:** branched-chain amino acids; **VO2peak:** peak oxygen consumption; **Kg:** kilogram; **g:** gram; **DAPA:** dapagliflozin; **IR:** insulin resistance; **HbA1c:** hemoglobin A1c; **PRISMA:** Preferred Reporting Items for Systematic Reviews and Meta Analyses; **PROSPERO:** International Prospective Register of Systematic Reviews; **SD:** standard deviation; **MRI:** magnetic resonance imaging; **MRS:** magnetic resonance spectroscopy; **CT:** computed tomography; **PEDro:** Physiotherapy Evidence Database; **CMA:** Comprehensive Meta-analysis; **SMD:** standardized mean difference; **WMD:** weighted mean difference; **CIs:** confidence intervals.
